# A Review of Generic Frameworks and Scales for Measuring Quality of Life and Well-Being: Toward Finding the Right Ruler

**DOI:** 10.3390/bs16050629

**Published:** 2026-04-23

**Authors:** Mohamad Adam Bujang

**Affiliations:** Clinical Research Centre, National Institutes of Health, Sarawak General Hospital, Ministry of Health Malaysia, Kuching 93586, Malaysia; mohamadadambujang1980@gmail.com

**Keywords:** QOLS, SigQOLM, WHOQOL-BREF, quality of life, well-being

## Abstract

Assessing quality of life (QOL) and well-being ideally requires considering a broad range of measurable factors. This article reviews the various frameworks of QOL and well-being published in the literature, with the aim of comparing their domains. Moving forward, this study elaborates on the justifications and applications of the recent scale. The previously established QOL and well-being scales identified include the Quality of Life Scale (QOLS), WHOQOL-BREF, and the Significant Quality of Life Measure (SigQOLM). A total of 41 domains were compared and discussed. The WHOQOL-BREF (13/26 = 50.0%) and SigQOLM (33/69 = 47.8%) incorporate a balanced item set that measures both health and non-health elements. In SigQOLM, the majority of indicators for QOL and well-being (39/41) are represented with more than one item. Thus, SigQOLM provides a more comprehensive framework for assessing QOL and well-being compared to earlier scales. Therefore, researchers, policymakers, and healthcare providers can utilize SigQOLM to gain a deeper understanding of the broader implications of healthcare and policy interventions on people’s lives.

## 1. Introduction

Every individual seeks to attain the highest possible quality of life (QOL) and well-being, recognizing these as fundamental elements for personal fulfillment. Governments and non-governmental organizations invest considerable resources aimed at improving QOL and well-being across different dimensions (DeGuimaraes et al., 2020; Mo et al., 2022). With such importance placed on QOL and well-being, individuals may ponder how to evaluate and quantify whether their lives are deemed ‘excellent’ or not.

Traditionally, two approaches are commonly used in evaluating the QOL and well-being of individuals. The first method relies on statistical indicators devised by economists, epidemiologists, and methodologists to assess the performance of a country or state, while the second approach involves gathering self-reported outcomes from individuals (B. C. Liu, 1976; WHOQOL Group, 1996, 1998). Throughout history, metrics such as life expectancy, literacy rates, per capita income, and mortality and morbidity figures have been widely employed to formulate various well-being indices. Many nations worldwide commonly utilize statistical indicators like the Consumer Price Index (CPI), foreign direct investment (FDI), inflation rate, and Statistical Capacity Indicators to depict a country’s performance, indirectly reflecting the QOL and well-being of its populace.

Nonetheless, there is a consensus that such measures or statistics are inadequate in capturing the entirety of well-being (Bujang et al., 2023; Colquhoun, 2011; WHOQOL Group, 1998). The reason for this is that these statistical measures or indexes may not be suitable for capturing individuals’ personal experiences of their QOL and well-being accurately. While these indicators may be suitable for assessing the performance of states or countries, they lack the depth needed to evaluate QOL and well-being adequately. Hence, the development of a validated scale measuring QOL and well-being becomes necessary. Therefore, this study aims to review generic frameworks and scales for measuring QOL and well-being. Moving forward, this study elaborates on the justifications and applications of the recent scale.

## 2. Materials and Methods

A comprehensive literature review was conducted to examine established generic frameworks and widely used quality of life (QOL) and well-being instruments. The review followed predefined inclusion criteria to ensure methodological rigor. Studies were included if the scales (i) presented a generic, rather than disease-specific, QOL or well-being framework or scale, and (ii) were published in peer-reviewed journals. Any study or scale that did not meet these conditions was excluded. Applying these criteria, three scales were identified: the Quality of Life Scale (QOLS), the WHOQOL-BREF, and the recently developed Significant Quality of Life Measure (SigQOLM). Overall, this study identified 41 distinct domains. Particular emphasis is placed on the rationale and applications underlying the newly developed comprehensive generic framework, which aims to advance the measurement of QOL and well-being across diverse populations.

## 3. Results

### 3.1. Literature Review

There are limited scales available to measure quality of life (QOL) and well-being. The earliest scale reported in the literature was the Quality of Life Scale (QOLS), developed in the late 1970s (Flanagan, 1978, 1982). QOLS is a 15-item instrument that measures five conceptual domains of quality of life, including material and physical well-being, relationships with other people, social, community and civic activities, personal development and fulfillment, and recreation. This scale uses a Likert scale of seven for responses. Out of the 15 items, only one specifically asks about health in general. This limitation of QOLS is noteworthy since the health domain, considered the most important aspect of well-being, should ideally require more items for measurement (Bujang et al., 2023; WHOQOL Group, 1998).

The earlier construct of QOLS was designed based on a conceptual framework and content validity. Probably due to its development as a classical scale in the late 1970s, elements of validity were not emphasized. In other words, initially, the construct of QOLS was not validated by common statistical techniques such as exploratory factor analysis and model fit. However, subsequent studies have attempted to validate its construct validity, yielding varying results (Burckhardt et al., 2003; Reeves et al., 2020). Nevertheless, the scale remains relevant and has been used in research up to the present day (Pearson et al., 2024; F. J. Wang et al., 2024).

On the other hand, WHOQOL-BREF, one of the well-known measures for assessing QOL and well-being, was developed by the WHOQOL Group. This work aimed to create a cross-culturally applicable quality of life assessment. Derived from the original WHOQOL questionnaire, which comprised 100 items, the WHOQOL-BREF was streamlined to 26 items. It encompasses four domains: physical health, psychological well-being, social relationships, and environment (WHOQOL Group, 1998). The WHOQOL-BREF includes the domain “Environment,” which measures several well-being indicators.

The SigQOLM measures a comprehensive spectrum of measures of QOL and well-being, encompassing 4 elements, 18 domains, and 69 items. The main pillars known as elements for SigQOLM are: “health with nine domains,” “relationship with 3 domains,” “functional activities with 3 domains,” and “survival with 3 domains.” On another note, the SigQOLM includes many items, which could be considered as one of its limitations. Thus, it is imperative for future studies to streamline the questionnaire by reducing the number of items. This would not only shorten the time required for survey completion but also facilitate ease of participation, particularly among elderly respondents.

### 3.2. Comparisons

A summary of comparisons between different QOL and well-being scales is presented in Table 1. SigQOLM covers almost all items or domains except for “Rearing children—found in QOLS” and “Sex life—found in WHOQOL-BREF.” The most important domain is health, and hence SigQOLM covers nine domains consisting of 33 items out of 69 items (47.8%). WHOQOL-BREF covers health based on two domains, which are “Physical health” and “Psychological health,” both with 13 items out of 26 items (50.0%). In contrast, QOLS only covers the health domain with one item out of 16 items (6.3%).

Most indicators in QOLS and WHOQOL-BREF are represented by a single item. For example, family relationships are represented by item 3 in QOLS and item 20 in WHOQOL-BREF, whereas SigQOLM uses four items (items 1 to 4 in the Relationship dimension) to measure the same indicator. In addition, SigQOLM measures relationships, functional activities, and survival, spanning nine domains, with each domain measured by three to five items. The unique QOL and well-being indicators in SigQOLM, which are not measured in QOLS and WHOQOL-BREF, are “Eating regime,” “Perception of future health,” “Religious,” and “Perception of future conditions.”

## 4. Discussion

The motivation to develop SigQOLM was not only driven by the fact that previous scales were developed more than 20 years ago. More importantly, SigQOLM was developed to address the limitations present in those previous scales. For example, construct validity for QOLS was not measured during the development phase. On the other hand, most domains in WHOQOL-BREF are measured by a single item. For instance, within the “health” domain, it only addresses sleep quality and body image with one item each, despite there being various aspects to consider regarding a person’s satisfaction with these factors.

These health indicators, such as sleep quality and body image, could be further rephrased within the same domain to develop more comprehensive and fair measures (Cox et al., 2010; Nayir et al., 2016; Stojanov et al., 2020; Zeitlhofer et al., 2000). In comparison, SigQOLM also measures “eating regime” and “perception of future health.” Eating regime is an important health indicator since patients with chronic diseases such as diabetes mellitus may need to alter their dietary regimes, inevitably affecting their quality of life (S. F. Abraham et al., 2006; Cerrelli et al., 2005; Jenkins et al., 2011). Furthermore, perceptions of future health conditions can be disrupted by uncertainties when a person is affected by a rare pandemic like COVID-19 (Ahorsu et al., 2020).

Unlike WHOQOL-BREF, SigQOLM does not include items for assessing sexual life, although this aspect may be crucial for QOL and well-being. It is important to acknowledge that not all individuals are married, especially adolescents, the widowed, or the divorced, who may not have a sex life initially. Additionally, SigQOLM does not recognize sexual relationships outside of official partnerships, such as with other women or others’ spouses, as indicators of QOL and well-being. For this reason, SigQOLM did not incorporate any domains or items related to sexual life to avoid inadvertently introducing ‘missing values’ and inappropriate findings (Bujang et al., 2018, 2023). Therefore, addressing a healthy sex life may necessitate adopting alternative approaches or developing a completely different set of questionnaires for this purpose (L. Abraham et al., 2018).

WHOQOL-BREF and SigQOLM do not include rearing children, which is measured in QOLS. Although raising children is undeniably an important and meaningful aspect of life, not everyone has the opportunity to do so. For young adults and older individuals, this aspect may also be less relevant. Since SigQOLM is designed as a generic scale applicable to almost all adults, this domain was intentionally excluded. This decision also helps to minimize the risk of excessive missing data, which could otherwise compromise the scoring mechanism (Bujang et al., 2018, 2023).

SigQOLM comprises 4 elements, 18 domains, and a total of 69 items, providing a more comprehensive approach to assessing QOL and well-being compared to earlier scales. Its elements include health, relationships, functional activities, and survival. In other words, an individual with excellent QOL and well-being is one who enjoys optimal health, fosters positive relationships, is highly capable of performing diverse functional activities, and demonstrates excellent survival in life. SigQOLM was validated using Exploratory Factor Analysis (EFA) and Confirmatory Factor Analysis (CFA), and the results showed excellent construct validity with moderate to strong factor loadings, model fit, and internal consistency (Bujang et al., 2023). It is open-source, allowing anyone to use it for free, with the goal of increasing equity in access to the tool to improve the QOL and well-being of people.

This article also recommends methods of analysis when using SigQOLM. Besides descriptive analysis in determining excellent or poor QOL and well-being, researchers can determine the factors associated with overall QOL and well-being or with each respective element and domain (Bujang et al., 2015; Wen et al., 2014). The original scores are in numerical form. Researchers can use cut-off scores to differentiate between “excellent to good” versus “moderate to poor” conditions (Bujang et al., 2025). Hence, logistic regression can be used for regression analysis.

### 4.1. Applications

In the pursuit of understanding human well-being and assessing the effectiveness of interventions aimed at enhancing the quality of life, the development and application of SigQOLM stand as a pivotal milestone. This scale serves as a comprehensive tool for evaluating various dimensions of an individual’s life, encompassing health, relationships, and survival factors (Bujang et al., 2023). Through its application, researchers, healthcare professionals, and policymakers can gain valuable insights into the holistic nature of human flourishing and design strategies to optimize it.

One significant application of SigQOLM lies within the field of healthcare. In clinical settings, healthcare providers utilize the scale to assess the impact of medical treatments on patients’ overall quality of life (Araujo et al., 2016; Bozkurt & Yılmaz, 2016; Skevington, 1998). By measuring changes in different domains before and after interventions, practitioners can tailor treatment plans to not only address specific health issues but also improve patients’ overall well-being. For individuals facing chronic illnesses, the health dimension in SigQOLM offers a means to track their subjective experiences and adjust care plans accordingly, fostering a patient-centered approach to healthcare (Bujang et al., 2024).

The application of SigQOLM in building relationships offers a structured approach to understanding and nurturing the well-being of individuals within interpersonal connections and religious life. By incorporating this scale into relationship assessments, individuals can systematically evaluate various aspects of their shared experiences, including emotional satisfaction, social support, and religious life (Bennich et al., 2019; Jakobsson & Hallberg, 2005; Modanloo et al., 2019; Moon & Kim, 2013; Rule, 2017; Ware & Sherbourne, 1992; WHOQOL Group, 1998). Utilizing SigQOLM in interventional studies that aim to foster relationships allows researchers to identify strengths and areas for improvement in a specific community.

In addition, the application of SigQOLM in assessing functional activities provides a systematic framework for enhancing individuals’ engagement and satisfaction in daily tasks and routines. By incorporating this scale into assessment, researchers can evaluate self-care, social life, and perception of time usage, and this information is useful for assessing the impact of various factors on individuals’ quality of life (Gill et al., 2013; W. C. Wang et al., 2011; WHOQOL Group, 1998).

Furthermore, the application of the “survival” dimension serves as a crucial tool for individuals facing challenging circumstances, such as safety concerns, socioeconomic crises, and uncertainties regarding future conditions (Albouy et al., 2014; Carr et al., 2001; Gou et al., 2018; McKee et al., 2015; Rosinha et al., 2018). By systematically assessing various domains of survival, SigQOLM empowers individuals to prioritize their needs and make informed decisions in survival situations. Moreover, healthcare providers, government agencies, and researchers can utilize the scale to prioritize interventions and allocate resources effectively, ensuring that individuals have the support they need to survive and recover (DeGuimaraes et al., 2020).

Beyond individual assessments, SigQOLM also serves as a valuable tool for informing public policy and social programs. By gathering data on the QOL and well-being of different populations, policymakers can identify disparities and allocate resources more effectively (X. Liu et al., 2023; Nakamura et al., 2022). For instance, communities experiencing high levels of poverty or environmental degradation may require targeted interventions to improve residents’ quality of life. By incorporating SigQOLM into policy evaluations, decision-makers can assess the impact of interventions over time and make evidence-based adjustments to promote societal well-being.

**Figure 1:**
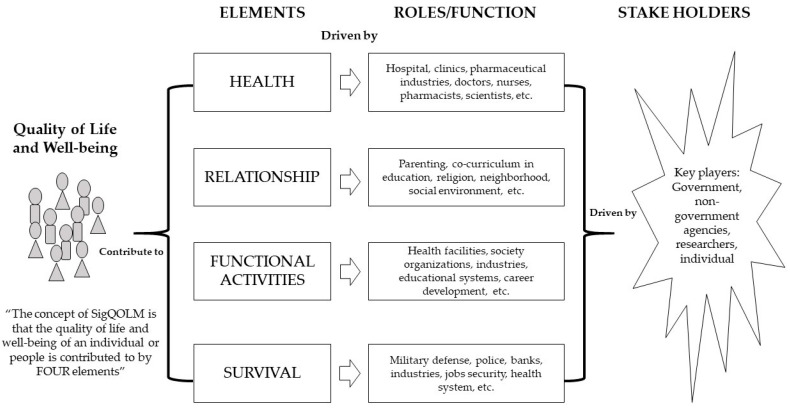
The illustration of SigQOLM and its relationship with people.

The comprehensive integration of QOL and well-being with people, roles/functions, and stakeholders is depicted in Figure 1. As previously discussed, the essence of SigQOLM lies in advancing the quality of life for individuals and communities, anchored on four fundamental pillars: health, relationships, functional activities, and survival. For instance, the health domain necessitates collaboration among hospitals, clinics, pharmaceutical industries, medical practitioners, and researchers to ensure optimal levels of well-being and vitality. Initially, these initiatives stem from concerted efforts by governmental and non-governmental entities. Hence, governmental bodies, non-governmental organizations, and individuals must formulate strategies, programs, interventions, and policies to address health-related challenges, ultimately enhancing the overall quality of life.

The underlying principle is straightforward. All actions undertaken by governmental bodies, non-governmental organizations, and individuals, whether as singular entities or organized groups, are geared towards enhancing the quality of life and well-being of the populace. These efforts converge upon four primary elements, as previously elucidated, constituting the foundational requisites for individuals’ well-being. While acknowledging the significance of wealth and recognition, SigQOLM posits that these factors do not serve as prerequisites for quality of life and well-being. Although individuals may aspire to financial success and acclaim, their attainment does not guarantee exemplary quality of life if essential elements such as health, relationships, functional activities, and survival are compromised.

Recognizing that the pursuit of wealth and recognition often yields unsatisfactory outcomes for many (i.e., not everyone can become rich and famous), SigQOLM refrains from considering these pursuits as indicators of quality of life and well-being. Instead, by prioritizing and optimizing the essential elements, governmental and non-governmental bodies, alongside individuals, can undertake concerted efforts to bolster these aspects and evaluate progress using pertinent metrics. Consequently, the development of SigQOLM is intended to promote research and initiatives aimed at enhancing quality of life and well-being, thereby fostering an improved standard of living for all individuals.

### 4.2. Important Note

The primary motive of this study is to provide a comprehensive overview of the development and evolution of generic quality of life (QOL) and well-being scales. This represents an important innovation in the field, tracing the progression from the first widely recognized measure, the Quality of Life Scale (QOLS), to subsequent instruments such as the WHOQOL-BREF, and most recently, the Significant Quality of Life Measure (SigQOLM). Each of these scales offers unique strengths and has certain limitations. For example, the QOLS is particularly suitable when a very brief and rapid assessment is required, making it ideal for time-limited surveys or large-scale screenings. The WHOQOL-BREF, on the other hand, is often preferred for studies requiring comparison across different populations and research settings, due to its robust cross-cultural validation. Finally, the SigQOLM provides a more holistic and comprehensive measurement of quality of life and well-being, capturing multiple dimensions that may not be fully addressed by earlier instruments. By highlighting these distinctions, the study aims to guide researchers, practitioners, and policymakers in selecting the most appropriate tool for their specific objectives and contexts.

### 4.3. Strengths and Limitations

The strength of this study lies in its portrayal of the history, development, and progress of a generic framework for QOL and well-being over more than three decades. Life circumstances and how people perceive life and QOL may also have evolved during this period. However, since the scope of this study focuses solely on reviewing generic frameworks of QOL and well-being, only three well-known scales were included. This limited number of scales for comparison may, in turn, restrict the diversity of the discussion.

## 5. Conclusions

SigQOLM offers a more comprehensive framework for measuring the QOL and well-being of people. This scale was developed with the motivation to improve people’s QOL and well-being across various important aspects of human life. The effort to measure people’s QOL and well-being is crucial for identifying which elements require support and for strategically allocating resources. This work promotes equal welfare for all individuals and helps eliminate discrimination based on region, gender, and race.

## Figures and Tables

**Table 1:** Summary of comparisons between QOLS, WHOQOL-BREF, and SigQOLM.

		Quality of Life and Well-Being Scales		
No.	Domains/Items	QOLS	WHOQOL-BREF	SigQOLM
1	Physical pain		item 3	items 1 to 5: Dimension “Health”
2	Physical energy/Vitality		item 10	items 6 to 9: Dimension “Health”
3	Emotional symptoms		item 26	items 10 to 12: Health’ dimension
4	Independent	item 16		items 13 to 15: Dimension “Health”
5	Mobility		item 15	items 16 to 19: Health’ dimension
6	Sleep quality		item 16	items 20 to 23: Dimension “Health”
7	Eating regime			items 24 to 25: Dimension “Health”
8	Body image		item 11	items 26 to 29: Dimension “Health”
9	Perception of future health			items 30 to 33: Dimension “Health”
10	Family	item 3	item 20	items 1 to 4: Relationship dimension
11	Friendship	item 6	item 22	items 5 to 8: Dimension Relationship
12	Religious			items 9 to 12: Dimension Relationship
13	Self-care		item 17	items 1 to 5: Dimension “Functional activities”
14	Social life	item 13	items 20 and 22	items 6 to 8: Dimension “Functional activities”
15	Perception of time usage (i.e., learning, work, activities)	item 11	items 17 and 18	items 9 to 13: Dimension “Functional activities”
16	Basic needs (i.e., home, financial, food, etc.)		item 12	items 1 to 3: Dimension “Survival”
17	Safety		item 8	items 4 to 7: Dimension “Survival”
18	Perception of future conditions			items 8 to 11: Dimension “Survival”
19	Materials and physical well-being	item 1	Cover under domain “Environment”	Cover under element “Health” and “Survival”
20	General health	item 2	item 2	Cover under Health-SigQOLM score
21	Active recreation	item 15	item 14	Cover under domain “Perception of time usage”
22	Entertainment/enjoy life	item 14	item 5	Cover under domain “Perception of time usage”
23	Expressing creativity	item 12		Cover under domain “Perception of time usage”
24	Understanding yourself (i.e., assets and limitations)	item 10		Cover under domain “Perception of future conditions”
25	Learn knowledge	item 9		Cover under domain “Perception of time usage”
26	Participate in community activities	item 8		Cover under domain “social life”
27	Helping or encouraging others	item 7		Cover under domain “Family relationship, Friendship, and social life”
28	Relationship with spouse	item 5		Cover under domain “Family relationship”
29	Having and rearing children	item 4		
30	General quality of life	Cover under QOLS score	item 1	Cover under SigQOLM score
31	Satisfied with yourself	Cover under QOLS score	item 19	Cover under SigQOLM score
32	Requirement for medical treatment		item 4	Cover under domain “Basic needs” and “Perception of future health”
33	Meaningful life	Cover under QOLS score	item 6	Cover under SigQOLM score
34	Able to concentrate		item 7	Cover under domain “emotional symptoms”
35	Physical environment	Cover under item 1	item 9	Cover under domain “Basic needs” and “Perception of future conditions”
36	Satisfaction with yourself	Cover under QOLS score		Cover under SigQOLM score
37	Living place	Cover under item 1	item 23	Cover under domain “Basic needs” and “Safety”
38	Access to health’s services		item 24	Cover under domain “Basic needs” and “Perception of future health”
39	Transportation	Cover under item 1	item 25	Cover under domain “Basic needs”
40	Accessibility to information		item 13	Cover under domain “Basic needs” and “Perception of future conditions”
41	Sex life		item 21	

## Data Availability

No new data were created or analyzed in this study. Data sharing is not applicable to this article.

## Abbreviations

The following abbreviations are used in this manuscript:

CPI	Consumer Price Index
FDI	Foreign Direct Investment
QOL	Quality of Life
QOLS	Quality of Life Scale
SCI	Statistical Capacity Indicators
SigQOLM	Significant Quality of Life Measure
WHOQOL-BREF	World Health Organization Quality of Life-BREF
